# Experiences and outcomes of older adults with obesity transitioning from gym- to home-based resistance training due to COVID-19 lockdowns: a mixed-methods analysis of a RCT

**DOI:** 10.1186/s12877-025-06247-3

**Published:** 2025-07-29

**Authors:** Costas Glavas, Jakub Mesinovic, Anoohya Gandham, Mavil May Cervo, Carrie-Anne Ng, Peter R. Ebeling, Elena S. George, Robin M. Daly, Belinda R. Beck, Paul Jansons, David Scott

**Affiliations:** 1 Institute for Physical Activity and Nutrition (IPAN), School of Exercise and Nutrition Sciences, Deakin University, Geelong, VIC Australia; 2 Department of Medicine, School of Clinical Sciences at Monash Health, Monash University, Clayton, VIC Australia; 3 Mary MacKillop Institute for Health Research (MMIHR), Australian Catholic University, Melbourne, VIC Australia; 4 School of Nutrition, Philippine Women’s University, Manila, Philippines; 5 Centre for Health Economics Research and Evaluation, University of Technology Sydney, NSW Sydney, Australia; 6 Griffith University, The Bone Clinic, Gold Coast, Coorparoo, QLD Australia

**Keywords:** Older adults, Obesity, Exercise, Weight loss, Experiences, Perspectives

## Abstract

**Background:**

Supervised gym-based high-intensity resistance and impact training (HiRIT) can enhance physical function and muscle strength, but older adults may face challenges affecting adherence to HiRIT, such limited access to facilities and lack of transportation, necessitating a shift towards unsupervised home-based exercise. The aim of this study was to explore experiences and perspectives of older adults with obesity who were required to transition from supervised gym-based HiRIT to unsupervised home-based resistance training (RT) and aerobic training (AT) during COVID-19 lockdowns. Secondary aims were to compare changes in body composition and physical function after 12 weeks between participants required to transition to home-based exercise (“HOME”) and those who were able to continue gym-based exercise (“GYM”).

**Methods:**

Thirty older adults (60–89 years) with obesity were enrolled from the gym-based HiRIT intervention arm of a 12-week exercise and dietary weight loss trial. Thirteen (43%) participants were transitioned to HOME due to COVID-19 lockdowns. HOME participants were prescribed bodyweight RT and AT exercises, while maintaining the weight loss intervention. Eight HOME participants completed semi-structured interviews post-intervention. Quantitative outcomes including exercise adherence, body composition and physical function were compared to GYM participants.

**Results:**

Participants’ experiences and perspectives regarding the HOME program encompassed various elements including accessibility, accountability, maintaining physical activity levels, motivation, support from health care professionals, openness to telehealth videoconferencing for support, engagement, lack of equipment, supervision and a structured routine. Both groups had significant reductions in body mass (mean ± SD; GYM: -4.4 ± 0.4 kg, HOME: -6.2 ± 1.2 kg), but HOME demonstrated greater losses in fat mass (mean difference: -3.1 kg, 95% CI: -6.0, -0.3) compared with GYM represented by a large effect size (d = 0.8). Physical function outcomes improved only in GYM (all *P* < 0.05).

**Conclusions:**

Older adults with obesity transitioning from supervised gym-based to unsupervised home-based exercise face both supportive and challenging experiences. While accessibility and accountability enhanced their engagement, some participants faced difficulties related to limited equipment and digital support, emphasising areas for improvement in home-based exercise interventions. Home-based exercise may be effective for supporting dietary weight loss, but further research is needed to determine if there are any beneficial effects on physical function.

**Trial registration:**

Australian New Zealand Clinical Trials Registry (ANZCTR) ACTRN12618001146280; date of registration: 12/07/2018.

**Supplementary Information:**

The online version contains supplementary material available at 10.1186/s12877-025-06247-3.

## Introduction

Lifestyle interventions, including structured physical activity programs and dietary modifications can improve physical function, body composition, and muscle strength in older adults with obesity [1–6]. Typically weight loss is associated with reductions in obesity-related comorbidities, such as cardiovascular disease, type 2 diabetes and mobility impairments [7–9]. However, intentional weight loss alone is often discouraged for older adults with obesity due to the concomitant lean muscle mass losses that occur [10, 11], which can range from 10 to 50% of total tissue loss depending on the magnitude of weight loss [12, 13]. While increasing physical activity levels can decrease risk of comorbidities and increase functional benefits, supervised, high-intensity resistance and impact training (HiRIT) may offer a more comprehensive strategy by concurrently reducing adiposity while preserving muscle and bone mass, improving physical function and preventing progressive chronic diseases in older adults with obesity [14, 15].

HiRIT has demonstrated promising outcomes in older adults, including improvements in muscular strength, bone density and physical function [16–18]. However, its implementation often requires access to specialised gym equipment and supervision to ensure safety and adherence. During the initial stages of the COVID-19 pandemic, older adults were required to isolate, reducing their access to gym-based lifestyle programs [19]. A systematic review conducted in 2021 including 66 studies highlighted a > 50% decrease in physical activity during lockdowns, accompanied by increased sedentary behaviour in adults with and without chronic conditions [20]. Another study of active older adults who were impacted by lockdowns found there was a significant ~ 50% decrease in self-reported physical activity levels [21].

Transitions from traditional gym-based to home-based exercise interventions were necessitated by COVID-19 lockdowns and may now represent a feasible approach to support older adults with obesity to engage in exercise programs [22]. Beyond COVID-19 lockdowns, the convenience and flexibility of home-based exercise programs, in some cases supported by improvements in digital health platforms, have facilitated engagement among older adults [23, 24]. Home-based exercise programs may reduce physical inactivity and have been reported to be acceptable and feasible as they alleviate challenges associated with gym-based exercise programs such as a lack of access to transportation and facilities and associated costs in older adults [25]. Although, much of the evidence pertains to increase general physical activity or moderate intensity resistance training rather than high-intensity programs such as HiRIT [26, 27]. Furthermore, there may be safety concerns for older adults with obesity who often require higher levels of supervision to prevent injuries and support to avoid discontinuation of exercise training [28, 29]. It remains unclear whether transitioning from supervised gym-based exercise to unsupervised home-based exercise is acceptable, feasible and maintains clinical effectiveness for older adults with obesity. Additionally, little is known about the experiences and perspectives of older adults with obesity as they navigate this transition, including the challenges they, their motivations to continue training, and how they perceive the effectiveness and safety of home-based exercise programs.

The primary aim of this mixed methods analysis of a 12-week randomised controlled trial (RCT) of resistance and impact training and weight loss impacted by COVID-19 lockdowns was to explore experiences and perspectives to unsupervised, home-based exercise in older adults with obesity after transitioning from supervised gym-based training. Secondary aims were to evaluate differences in body composition, physical function, and physical activity for participants who transitioned to unsupervised home-based resistance training and aerobic training (RT + AT) (HOME group), compared with participants whose supervised gym-based HiRIT intervention was not interrupted by lockdowns (GYM group).

## Materials and methods

### Study design and participants

This study is a mixed methods analysis of a 12-week pilot RCT [30] of 60 older adults (aged between 60 and 89 years) with obesity (a body fat percentage ≥ 30 [men] or ≥ 40 [women] determined by dual-energy X-ray absorptiometry [DXA]) [31] and a mobility impairment (Short Physical Performance Battery (SPPB) score of ≤ 11) with the primary outcome being to assess improvements to gait speed. Participants were recruited into the RCT through electronic advertisements posted on websites and social media, and hard copy advertisements posted on noticeboards at Monash Medical Centre (Clayton, Victoria, 3168), local general practices and community centres. Participants were ineligible if they resided in a nursing home; were unable to walk 400 m in 15 min without the use of walking aids; were non-English speaking or had difficulty communicating; had moderate or severe cognitive impairment (defined as a Mini-Mental State Exam score of less than or equal to 18 points out of 30) [32]; undertook 4 or more weeks of supervised exercise or dietary targeted at weight loss or strength training in the past 6 months; planned to be away for 4 weeks or more during the intervention; had any self-reported neurological disorders; had self-reported severe knee or hip osteoarthritis; had any self-reported cardiovascular or cardiopulmonary disease; or any other disorder of such severity that life expectancy was less than 12 months. Participants in the original RCT completed a screening appointment at Monash Medical Centre (Clayton, Victoria, 3168), where they underwent the SPPB, a mini-mental state exam, a 400 m walk test and an assessment of their body fat percentage. Written informed consent was obtained from all participants prior to data collection.

Following screening measurements, eligible participants were randomised (computer-generated block randomisation) to either supervised gym-based high-intensity resistance and impact training (HiRIT) or home-based aerobic training with both groups undergoing a dietary weight loss intervention. The full methods, protocol, outcomes and analysis of the original RCT have been described previously [30]. This secondary analysis is a mixed-methods study which included only participants randomised to the HiRIT group. The trial commenced in 2019 and at the initiation of the first COVID-19 lockdowns in Australia in March 2020, and over six subsequent lockdowns between 2020 and 2021, existing HiRIT participants transitioned to unsupervised home-based RT + AT (described below).

This study adheres to the CONSORT guidelines for reporting clinical trials (Supplementary File 3), was approved by the Monash Health Human Research Ethics Committee (HREC/18/MonH/399) and was prospectively registered with the Australian New Zealand Clinical Trials Registry (ANZCTR; Trial ID: ACTRN12618001146280; date registered 12/07/2018).

### Dietary intervention

During the 12-week intervention period, all 30 participants followed the dietary weight loss intervention. The dietary intervention aimed for a 750–1000 kcal caloric deficit aiming for 0.5–1.0 kg reduction in total body fat mass per week and was prescribed by an accredited practising dietitian (APD). Furthermore, supplementation of protein (whey protein isolate; ~30 g per serve), vitamin D (cholecalciferol; 1000 IU/day) and calcium (600 mg/day) was provided to all participants with the aim of ensuring adequate intakes of nutrients for maintenance of musculoskeletal health while consuming a hypocaloric diet. Participants completed a self-reported three-day food record at baseline and the 12-week follow-up, which were validated by the APD through a one-on-one interview with the study participant at each time point. At the start of the intervention, each participant was oriented by the APD on how to quantify their intakes using food models and measuring utensils, as well as on how to reduce food portion sizes and replacing energy-dense foods with those of lower energy density. Furthermore, the APD conducted telephone interviews every week throughout the intervention to monitor and review dietary intakes and supplement compliance.

### High-Intensity resistance & impact training (HiRIT)

Participants allocated to the HiRIT group were prescribed a structured 12-week, twice-weekly, 30-minute, supervised, gym-based HiRIT program. All participants were encouraged to attend each supervised session run by an accredited exercise physiologist (AEP) at a local gymnasium, Healthwise Fitness, at Monash Medical Centre, Clayton. The prescribed exercise program used an Olympic bar, dumbbells and/or weight plates unless contraindicated. All participants were individually prescribed four fundamental exercises (deadlift, overhead press, and back squat and modified jumping chin-ups) throughout the intervention period. Participants performed up to 2 sets of 5 repetitions of all four exercises at 50% of 1 Repetition Maximum (RM) to serve as a warm-up at each session as required. Intensity was determined by 1 RM testing at the beginning and the end of the intervention. Participants would perform 5 sets of 5 repetitions, at an intensity of > 80–85% 1 RM at each HiRIT session. Each participant was encouraged to increase the load of the four prescribed exercises each session while maintaining the desired intensity if able. 1 RM intensity was modified weekly by study staff by determining the maximum amount of weight that could be performed for 1 set of 5 repetition, if participants were able to complete this set than their weights would be increased in the following week. During each session, investigators filled in an exercise diary to assess participants’ progression and to monitor adherence throughout the exercise intervention.

### Home-based resistance training & aerobic training (RT + AT)

Participants affected by COVID-19 lockdowns (Fig. 2) were provided with a modified, home-based unsupervised RT + AT program prescribed by an AEP to follow at home with written instructions to complete the exercises and use weights if they were available to them. The exercises were personalised by the AEP and consisted of strength exercises (calf raises, squats, wall or knee push-ups) and aerobic activity (brisk walking). Participants were instructed to complete two exercise sessions per week and depending on the participant’s fitness levels the AEP individually prescribed varying sets and repetitions for the calf raises, squats, and wall/knee push-ups (ranging from 3 sets by 5 repetitions to 5 sets by 15 repetitions). This modified program was deemed by the investigators to be safe and feasible for the participants to complete while unsupervised in their homes. Participants were also instructed to complete a minimum of 20 min of brisk walking per exercise session. Aerobic exercise was prescribed in addition to the RT to align with the recommendation of 150 min per week of physical activity by the Australian guidelines for physical activity in older adults. Participants in the HOME group were monitored and contacted weekly by study investigators via email to assess their progression with the RT + AT and if necessary, participants could call study investigators if they required further clarification on their home-based RT + AT.

### Adherence

Adherence was calculated as the number of completed sessions at the gym for supervised HiRIT in the GYM and the HOME groups divided by the total prescribed exercise sessions (24 sessions). Adherence to unsupervised home-based RT + AT was not recorded for the HOME group.

### Qualitative outcomes

The qualitative aspect of this study assessed the experiences and perspectives of older adults with obesity who transitioned from supervised gym-based HiRIT to unsupervised home-based RT + AT due to COVID-19 lockdowns. Thirteen participants were contacted via email to ascertain whether they would be interested in completing a phone interview at the conclusion of the study. Eight participants completed a one-on-one recorded, semi-structured, 10-minute phone interview with CG (study investigator) who has extensive experience in conducting qualitative interviews. The interviewer had no existing or ongoing relationships with the participants. Participants were aware of the purpose of the research and to our knowledge, no one else was present during the interview besides the participants and CG.

We used the Consolidated Criteria for Reporting Qualitative Research (COREQ) to report qualitative outcomes [33] and examined participants’ experiences and perspectives to unsupervised home-based exercise among older adults with obesity. The interview guide was developed by CG and PJ (study investigators) and consisted of 11 open-ended questions to elicit responses pertaining to participants overall experiences and perspectives of the program and their subsequent transition from supervised gym-based exercise to unsupervised home-based exercise (Supplementary File 2). CG asked further questions/prompted participants where necessary to clarify or obtain further information based on responses. All interviews were digitally voice recorded and were transcribed verbatim by a transcribing company (www.transcribeme.com TranscribeMe Inc.), after which data were deidentified. Interview recordings and transcripts were stored in a password-protected database, with only study investigators having secure access for a period of 15 years, after which it will be securely destroyed. Observations made by the researchers during and after the interviews were documented in field notes. No repeat interviews were carried out. Member-checking was implemented via email for the interview transcripts and a summary of the themes that were established to ensure we accurately represented participant experiences during the intervention. Data were imported into NVivo (version 14, QSR International Pty Ltd, Doncaster, Victoria, Australia) software for management and analysis.

### Quantitative outcomes

The following outcomes were assessed at baseline and 12 weeks:

#### Questionnaires and anthropometry

Participants completed self-administered questionnaires relating to their general demographics, overall health and health behaviours.

Body mass was measured with participants having fasted for a minimum of 12 h, with empty pockets, and with footwear, headwear, and heavy items of clothing (e.g. jackets), removed. Body mass was measured to the nearest 0.1 kg once only using electronic scales (Seca 804, Seca, Germany).

#### Body composition

Whole-body dual-energy X-ray absorptiometry (DXA) (Hologic Discovery A, Hologic, USA) scans were performed to determine whole-body fat mass and lean mass. The manufacturer’s spine phantom was used to calibrate the system on each scanning day and the short-term intra-individual CV for fat mass was 2.7% [34].

#### Physical function

##### Short Physical Performance Battery (SPPB)

The SPPB has been validated as a measure of physical performance and disability in older adults and is widely used in clinical and research settings [35]. A summary score of 0 to 12 (higher score indicating better function) was obtained based on performance in three tasks: chair rise test (also known as 5-time sit-to-stand test), standing balance assessments, and gait speed test.

##### Chair rise test

Participants began with their arms crossed over their chest in a standing position and were instructed to attempt to sit and stand as quickly as possible five times, without stopping in between. Study staff timed the test and counted the repetitions. The test was terminated if participants were unable to complete the 5 repetitions, used their arms for assistance, or took longer than 1 min to complete the test. Study staff stood close to the participant to provide support in the event that the participant may lose their balance. At the completion of the test, a score of 0–4 was given based on the time taken to complete the five repetitions.

##### Standing balance

These assessments consisted of three variations with differing difficulties: semi-tandem, full-tandem and side-by-side standing. Participants began with holding a semi-tandem stand for ten seconds, which involved placing the heel of one foot by the big toe of the other foot. Participants unable to hold this position would attempt the side-by-side position for ten seconds which involved standing with both feet touching side-by-side. Participants who completed the semi-tandem stand successfully were then instructed to hold a full-tandem stand for ten seconds, which involved placing their preferred foot directly in front of the other (touching heel-to-toes). Following the completion of the balance assessments, a score of 0–4 was given based on participant’s performance.

##### Gait speed test

A walking course 2.44 m length was used for this assessment. Participants were instructed to walk the course at their usual pace and the time taken to transverse the course was recorded. A score ranging from 0 to 4 was assigned based on completion time.

#### Hand grip strength

Hand grip strength was measured using a Jamar Plus digital hydraulic hand grip dynamometer (Patterson Medical, Bolingbrook, IL, USA) [36]. Participants were seated and instructed to extend their arm parallel to the ground and hold the instrument, then to grip and squeeze with maximal force for 3–5 s. Three tests were completed on each hand with 60 s rest times between each.

#### Stair climb test

Participants were instructed to climb a flight of 10 steps as quickly and as safely as possible, using the handrail for support if needed [37]. Once complete, participants walked back to the bottom of the staircase and were given a 60 s break. The test was repeated twice, and the average time taken to climb the steps across both trials was taken.

### Qualitative analysis

A modified thematic analysis based on the phases outlined by Braun and Clarke [38] was performed by CG. NVivo computer software (version 14, QSR International Pty Ltd, Doncaster, Victoria, Australia) was used to code, chart and map the data. Five stages of coding were completed: (i) Familiarisation; (ii) Identifying a thematic framework; (iii) Indexing; (iv) Charting; and (v) Mapping and Interpretation. Coding was initially completed deductively enforced by the interview questions developed by CG and PJ to elicit major themes from participants. Following this coding was completed inductively, depending on any new themes which arose through the interviews. An iterative process was used to test and retest the thematic framework. Two authors (CG and PJ) explored content and themes. Any disagreements were resolved by consensus moderation. CG and PJ reviewed and discussed the content, then refined the final themes to ensure they were within the context of the research question. The number of participants interviewed was based on data sufficiency (judged by reviewing transcripts after each interview and discussion between the researchers).

### Quantitative analysis

Quantitative data was entered and stored in a secure Microsoft Access Database and then exported into Stata SE 18 (StataCorp LLC, TX, USA) for analysis. Variables were inspected for data errors and in the case of missing or spurious data, original files were consulted. Any non-normal data was transformed to meet normality assumptions of parametric methods, otherwise non-parametric analysis was used where appropriate. Continuous descriptive variables were compared using independent sample t-tests if assumptions of normality (Shapiro-Wilk test) were met. For non-normally distributed variables, the Mann-Whitney U test was utilised. Categorical descriptive variables were compared using Chi-square tests. Exercise adherence (as a proportion of prescribed supervised exercise sessions completed in the gym) was compared between groups (GYM; *N* = 17, HOME; *N* = 13) using Chi-square tests. Independent sample t-tests and Mann-Whitney U tests were used to compare changes between groups in outcome measures of body composition, physical function between baseline and 12 weeks. Paired t-tests also compared changes within groups. Standardised effect sizes (Cohen’s d) were calculated for measures of body composition and physical function. For all analyses, statistical significance was defined as *P* < 0.05.

## Results

**Fig. 1:**
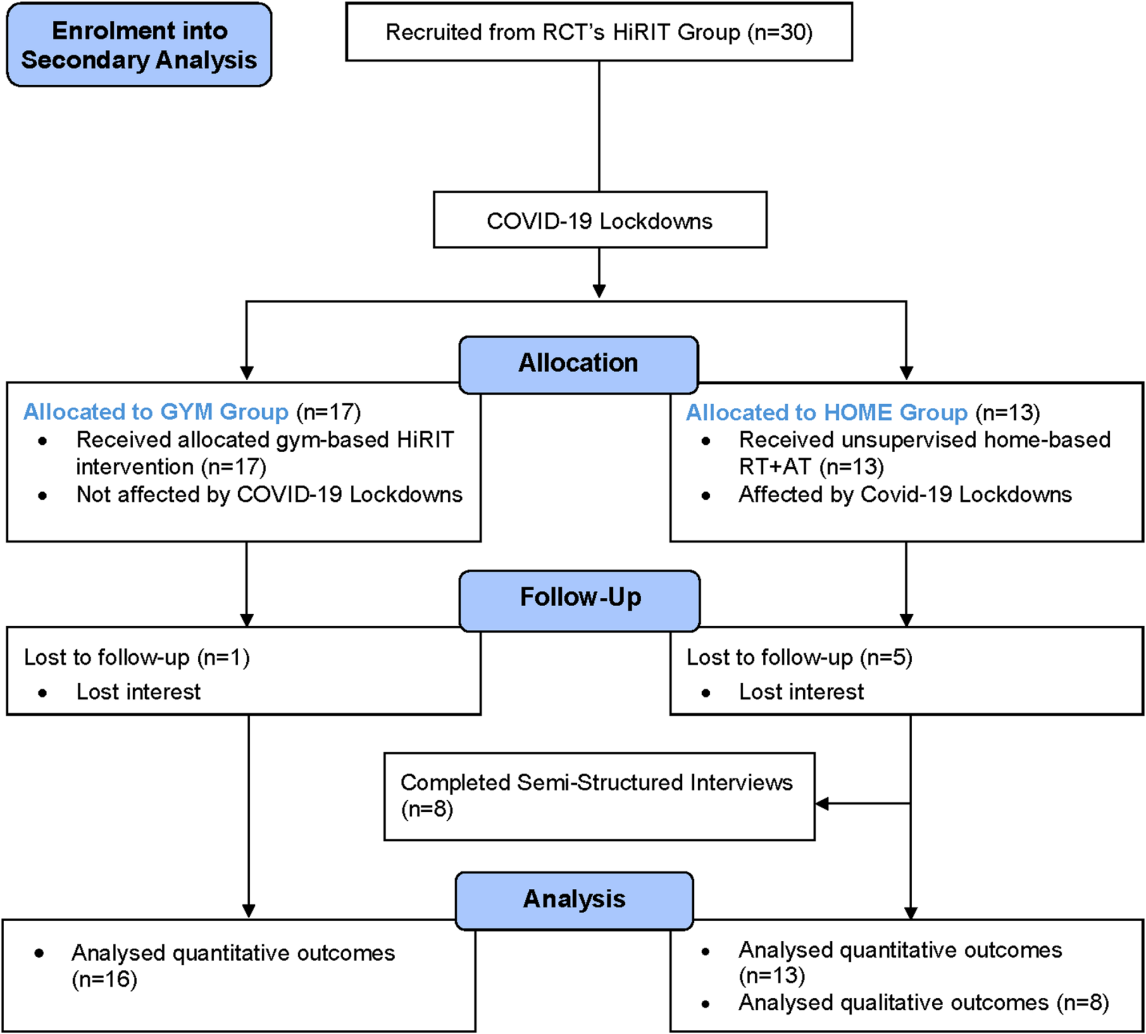
Consort flow diagram representing the flow of participants from the start to end of the study

**Fig. 2:**
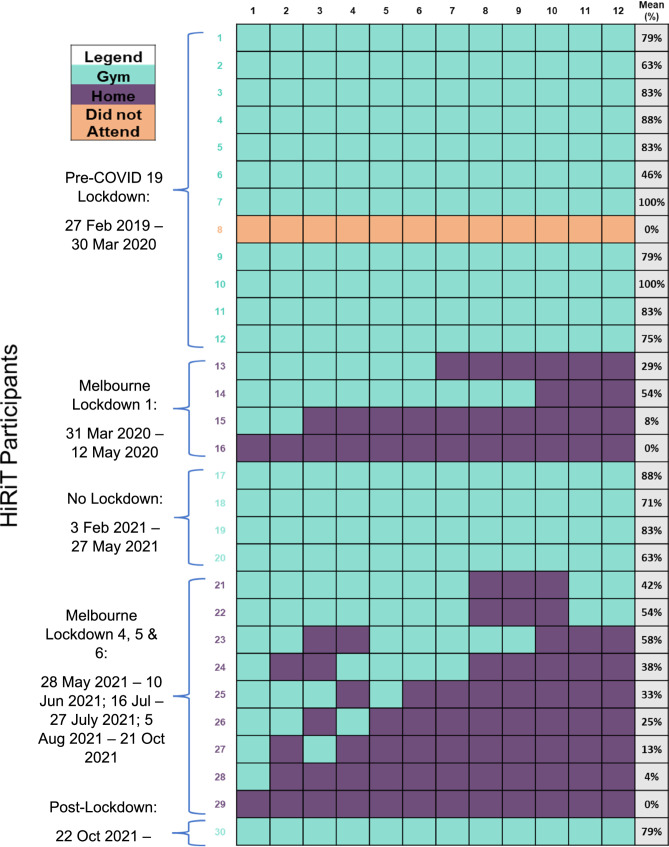
Heat map demonstrating participation in gym- and home-based exercise for individual participants with mean adherence (%) to training in the gym and completing supervised HIRIT *Participant 8 did not attend her intervention program due to an unrelated adverse event

The flow of participants throughout the study is depicted in Fig. 1. Thirty participants were initially randomised to supervised gym-based HiRIT, with 17 (57%) participants completing the gym-based program without the interference of COVID-19 lockdown (GYM). Thirteen (43%) participants were affected by the COVID-19 lockdown restrictions and transitioned to unsupervised home-based RT + AT (HOME). Due to multiple COVID-19 lockdowns, participants were interrupted for a varying number of weeks (HOME program < 4 weeks, *n* = 3; HOME program ≥ 5 to ≤ 9 weeks, *n* = 5; HOME program ≥ 10 weeks, *n* = 5) (Fig. 2). Eight HOME participants agreed to participate in a semi-structured interview at the conclusion of the study.

### Baseline demographics

Baseline participant demographics are presented in Table 1. Participants were aged between 60 and 89 years (mean ± SD age 69.5 ± 6.6 years). More than half of the participants were female and educated at a university level. Around half were non-smokers and almost all participants reported having at least one chronic health condition. There were no significant differences in baseline demographics between groups.

**Table 1:** Baseline demographics

	Intervention (Participants = 30)
Age – mean ± SD	69.5 ± 6.6
Gender (Female) – n (%)	19 (63%)
Parents Birthplace – n (%)^a^	
Australia	12 (41%)
Other	17 (59%)
Highest Level of Education – n (%)^a^	
Secondary/High School	6 (21%)
Tertiary or Further Educational Institute	6 (21%)
University or Other Higher Educational Institute	17 (59%)
Marital Status – n (%)^a^	
Single	4 (14%)
Widowed	3 (10%)
Divorced	2 (7%)
Separated	1 (3%)
Married or de facto	19 (66%)
Current Employment Status – n (%)^a^	
Employed Full-Time	5 (17%)
Employed Part-Time	8 (28%)
Retired	15 (52%)
Pension (including disability or sole pension)	1 (3%)
Smoker Status – n (%)^a^	
Ex-smoker	14 (48%)
Never Smoked	15 (52%)
Reported at least one chronic disease – n (%)^a^	27 (90%)

^a^*n*=29

### Adherence

Of the 13 HOME participants, eight spent six or more weeks undergoing unsupervised home-based RT + AT and two did not complete any supervised gym-based exercise (Fig. 2). The mean adherence to supervised HiRIT completed in the gym at 12-week follow-up by the GYM group was 74% (SD ± 5.6) compared to a mean of 28% (SD ± 5.1) for the HOME group.

### Qualitative outcomes

Ten key themes were identified from thematic analysis of the semi-structured interviews. Supplementary Table 1 presents the themes and illustrative quotes elicited from the thematic analysis on the experiences and perceptions of HOME participants.

**Fig. 3:**
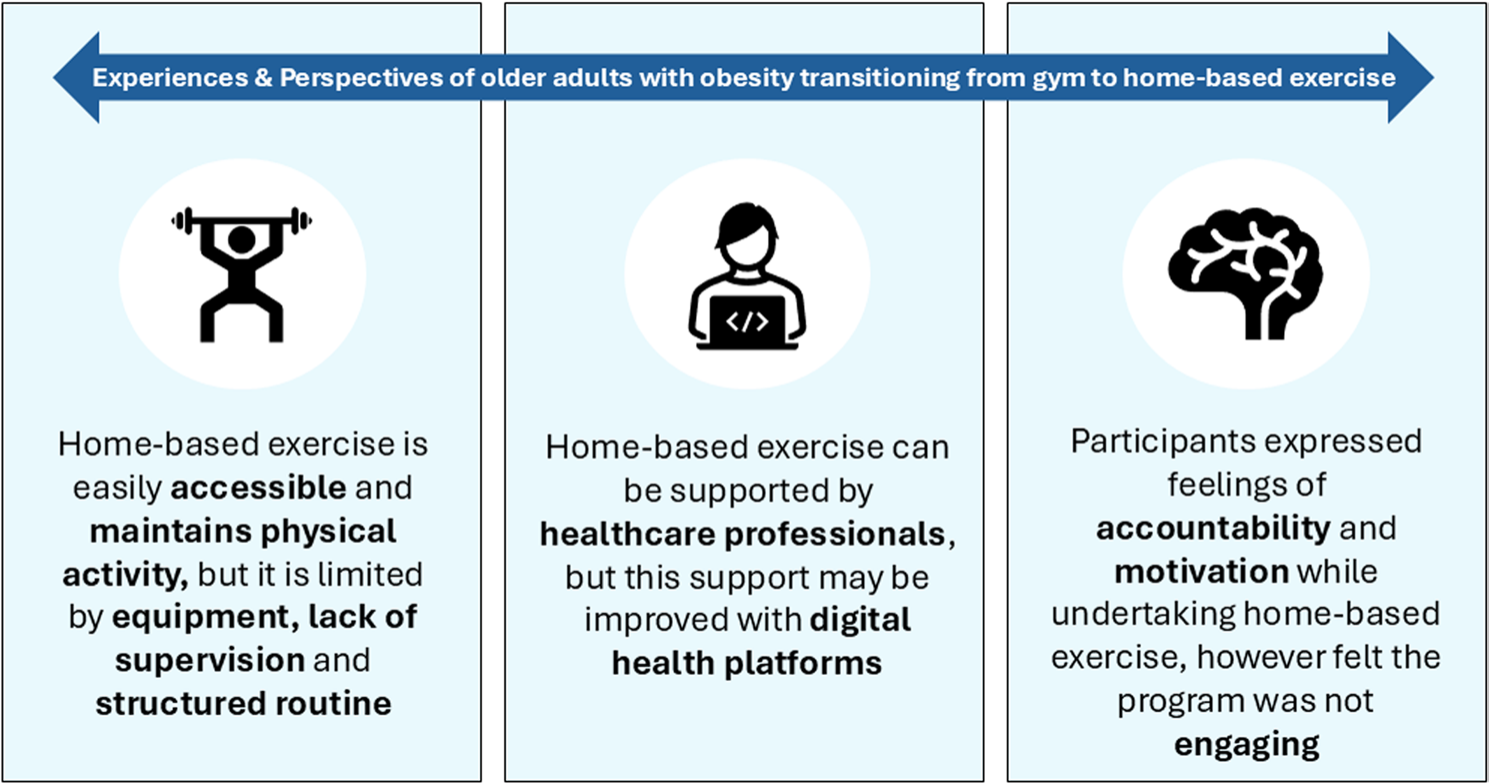
Salient themes extracted from the interview data regarding participants’ experiences and perspectives to unsupervised home-based exercise

Figure 3 presents the pertinent themes extracted from the interview data expressing participant’s feelings and motivations to different aspects of home-based RT + AT and the transition from gym- to home-based exercise. Participants’ experiences and perspectives regarding the HOME program encompassed both supportive and challenging aspects. They highlighted accessibility (participants could complete the intervention in the comfort of their own homes at any time which was convenient and they did not have to travel to community centres), accountability (participants felt an obligation to adhere to the study to the best of their abilities), maintaining physical activity levels, motivation, support from health care professionals and an openness to telehealth videoconferencing for support. Some participants also expressed challenges, including lack of engagement, equipment, supervision and structured routine.

#### Accessibility

Participants expressed positive sentiments about the accessibility of the home-based exercise which allowed them to be more flexible with their program and allowed them to be more efficient with their time-management. They were able to integrate the program into their daily activities, with participants noting, they felt they could access and complete the program at any time of day. The ability to exercise within a home environment also seemed to be a viable longer-term option for some participants as it reduced challenges presented by transportation and access to facilities.

“What worked better was if I —… was outside and I had a minute spare, I’d do a push-up against the fence, or I’d– … do my toe raises, you know, or my sitting up and down.” (Participant 49, female, age 63).

Additionally, with the restrictions of COVID-19 lockdowns, participants highlighted that the ability to complete home-based exercise was an excellent way to keep busy and stay active as they couldn’t leave their homes.

“Because we were locked down, it was, basically, one of the things that you could do” (Participant 30, female, age 70).

#### Accountability

HOME participants reported a sense of accountability in completing their prescribed exercise sessions, largely due to the requirement of check-ins with study staff to review their progress. Participants emphasised the importance of having someone inquire about their difficulties and needs, which assisted in reinforcing their commitment to the program. This regular intersection provided a supportive framework, which helped participants feel responsible for their progress.

“You know, … I’m still accountable, to some degree.’ …for someone to say, you know, ‘So how’s it going? Are you finding it difficult? You know, any way we– you know, anything you think we can do to help more?’” (Participant 48, female, age 64).

Some participants also highlighted the significance of making a commitment to study staff during the weeks they were training at the gym, which translated into a similar level of dedication to completing their home-based training. Further stating that by initially allocating the time for the gym-based exercise, they were able to maintain the allocation, and this assisted in maintaining adherence to the home-based exercise regimen.

“…if we made a commitment to the, um– for the gym guy– the actual gym attendant, then keep that commitment to do the exercise at home. Because I think, you know, most people would’ve allocated that time.” (Participant 54, male, age 68).

#### Support from healthcare professionals

HOME participants generally reported feeling supported by study staff and healthcare professionals while engaging in home-based exercise. Participants reported that having the ability to contact healthcare professionals at any time during the home-based intervention provided a strong sense of support and assurance. This continuous access to professional guidance helped mitigate any uncertainties or questions they had during their exercise regimen.

“And I had the backup and support of…the people there at Monash (the gym) …I was also supported because I could contact (the health professional) any time to ask any questions” (Participant 30, female, age 70).

Participants also highlighted regular check-ins and instructions from study staff contributed to their adherence to the home-based program. They acknowledged that for individuals in this population who may struggle with motivation to complete exercise, having healthcare professionals regularly assess and review their progress would assist in regulating adherence to home-based interventions.

“…Well, you sent me notes and asked me how I was going, so …I stayed on it. …So, I’m fairly self-motivated. I guess… if I was less motivated, I might have needed–…somebody to call in on the morning to see if I was going to do it. But, um” (Participant 52, female, age 63).

Notably accountability differed from the theme of support from healthcare professionals. While both contributed to adherence, accountability was externally driven, through an obligation to report to study staff. Whereas support was perceived as an available resource if guidance was needed from healthcare professionals.

#### Maintaining physical activity levels

Some HOME participants reported feeling capable of maintaining their physical function and activity levels through the transition from supervised gym-based to unsupervised home-based exercise. Participants emphasised that their involvement in the unsupervised home-based exercise allowed them to maintain muscle strength and mobility that they had achieved through the supervised gym-based program, which ultimately allowed them to return to the gym-based HiRIT without feeling they had regressed while unable to participate.

“(The HOME program) gave me the opportunity to maintain my strength and ability to… And I think every time I went back to the gym (after my participation in the study ended), I hadn’t– you know, I hadn’t regressed, which was good.” (Participant 48, female, age 64).

#### Motivation

A few HOME participants identified that they felt more motivated to complete the home-based exercises. Participants expressed that they were noticing progress while undertaking the home-based program and they felt their physical activity levels and mobility were improving, which in turn acted to increase their levels of motivation towards completing the program. Additionally, participants noted that participating in the HOME program assisted in establishing a routine, which improved their motivation and reinforced the sustainability of home-based exercise in the long-term.

“— (the HOME program) it really motivated me… And also, because I felt better, uh, it– I could feel myself getting better each week, it motivated me more.” (Participant 30, female, age 70).

#### Openness to telehealth videoconferencing for support

HOME participants reported that the introduction and implementation of digital interventions would be beneficial in delivering home-based exercise interventions. Participants highlighted that incorporating visual and audio communication software may be beneficial and could better reinforce feelings of commitment to the intervention and individualisation.

“Zoom conference would have been really good actually, especially if there was, um, you know, more than one (session).” (Participant 44, female, age 76).

“Keep that commitment, but then have a, you know, a Zoom or a teleconference or something to say, ‘Okay. Let’s, let’s run through it and let’s see how you’re going.’“(Participant 54, male, age 68).

These insights point to a common theme regarding the potential role of digital health and telecommunication in maintaining motivation and fostering feelings of support among older adults with obesity undergoing home-based exercise programs. The lack of digital modalities was expressed to be a challenge for participants when completing the home-based program. While there were check-ins by study staff via email, participants felt a lack of engagement and accountability without real-time face-to-face interaction. Telehealth and digital health modalities may be beneficial to increase engagement with home-based exercise, however modalities should also align with participant preferences, digital literacy and privacy concerns.

#### Lack of equipment

HOME participants identified not having access to gym equipment as a disadvantage to completing home-based exercise. Participants expressed there were difficulties in completing the HOME program while maintaining levels of intensity and physical exertion similar to that of the GYM program. They highlighted that replicating the GYM program without access to similar equipment was challenging and that some type of equipment to allow them to progress with exercises was needed.

“Yeah. The home-based exercise, it’s pretty hard when you don’t have access to, to those weights” (Participant 53, female, age 70).

#### Lack of supervision

HOME participants reported feeling that they needed some degree of supervision to complete the exercises correctly, efficiently and safely. They expressed that while undergoing direct supervision in the gym-based program they felt they were completing the exercises with proper form and efficiently, while they regarded the home-based program lacking this concept. Moreover, without study staff supervising their exercise session, there was a distinct lack of structure during the home-based program, compared to the gym-based sessions.

” You’re there, and they’re watching you directly and supervising you (in the gym), so I think obviously, that’s going to be better.” (Participant 30, female, age 70).

The lack of supervision for the home-based program led to a less disciplined approach to completing exercises. Furthermore, this led participants to be wary about whether they were completing the exercises correctly and safely at home. Some participants further expressed that it changed their attitude to adhering to the program as they were no longer supervised.

“--…there was no, um, uh, oversight, because, obviously, it was left up to me to do. And sometimes it was left up to me to do, I’d take a very laissez-faire attitude towards it.” (Participant 54, male, age 68).

Moreover, without oversight, some participants adapted a more passive approach to their exercise program, leading to disengagement with the program and decreasing their overall adherence.

#### Lack of engagement

Lack of engagement stemmed from participants feeling they were not invested in the program or lacked interest in completing the exercises while at home. Some participants identified that they could not engage with the home-based program, stating they found it repetitive and monotonous. They felt that continuously completing the same exercises at increased repetition was not feasible and they did enjoy it to the same degree as the GYM program.

“Yeah. But the repetitions, you know, doing 30-odd, what was it, calf raises or something, and– You know, it, it is monotonous doing it. Yeah.” (Participant 53, female, age 70).

Furthermore, the lack of social interaction was identified to play a role as participants stated that it was more difficult to engage with the HOME program as they were forced to complete it in isolation due to COVID-19 restrictions. The absence of supervision and face to face support, further contributed to disengagement with the program, particularly among the participants accustomed to structured training programs.

#### Lack of structured routine

Some HOME participants felt they could not find the time to complete the home-based exercise sessions as there was no commitment to meeting a schedule. They indicated that while working from home due to COVID-19 restrictions it was difficult to allocate time or have the energy to complete the program.

“I work strange hours. You know, running a house and stuff, it’s sort of hard-to-find time for yourself (to do the HOME program)” (Participant 48, female, age 64).

Participants described frequently postponing the program in lieu of other tasks and indicated that the lack of a set appointment schedule caused their adherence to the intervention to fall short. Participants expressed they would prefer a more individualised program which could accommodate for varying personal commitments and work requirements.

“Like every day I’d think, “Oh, I’ve got to do those. I’ll do them in an hour. No, I’ll do them in half an hour.” Sometimes it was like 3:00, 4:00 before I got around to it.” (Participant 53, female, age 70).

### Quantitative outcomes

#### Body composition and physical function

Table 2 summarises changes in secondary outcome measures at baseline and 12-week follow-up for GYM and HOME participants. Within-group analyses demonstrated that body mass, fat mass and lean tissue mass significantly decreased for both groups, but sit-to-stand time, SPPB score, and hand grip strength improved only in GYM. There were no significant differences between groups for changes in secondary outcomes, with the exception of fat mass which decreased significantly more in HOME compared with GYM and was represented by a large effect size (d=0.8).

**Table 2:** Baseline and 12-week values for secondary quantitative outcomes in GYM and HOME participants and p-values for the mean differences in change within and between groups

	**GYM**			**HOME**					
	**Baseline** **N=17**	**12-weeks N=17**	**Mean Change (95% CI)** **N=17**	**Baseline** **N=13**	**12-Weeks N=13**	**Mean Change (95% CI)** **N=13**	**Between Group Change (95% CI)**	**Between Group***P*-value	**Standardised Effect Size**
*Body Composition Outcomes*									
Body Mass (kg)	92.0 ± 16.5^1^	87.6 ± 16.1^1^	−4.5 (−5.8, −3.2)^1^	91.2 ± 12.5^2^	85.0 ± 10.3^2^	−6.2 (−10.4, −2.0)^2^	−1.7 (−1.4, 4.8)	0.266	0.3
Fat Mass (kg)	41.3 ± 8.9^1^	38.7 ± 8.8^1^	−2.6 (−4.0, −1.2)^1^	42.0 ± 8.7^3^	36.3 ± 6.2^3^	−5.7 (−9.1, −2.3)^3^	−3.1 (−6.0, −0.3)	*0.03*	0.8
Lean Mass (kg)	51.3 ± 10.5^1^	49.9 ± 10.6^1^	−1.4 (−2.3, −0.6)^1^	50.3 ± 10.3^3^	48.7 ± 9.6^3^	−1.6 (−3.0, −0.1)^3^	−0.1 (−1.4, 1.6)	0.866	0.03
*Physical Function Outcomes*									
Sit-to-Stand (sec)	10.5 ± 2.2^1^	9.1 ± 2.0^1^	−1.4 (−2.6, −0.2)^1^	9.5 ± 3.0^3^	8.4 ± 1.1^3^	−1.1 (−3.2, 1.0)^3^	−0.3 (−2.4, 1.8)	0.783	0.03
Gait Speed (m/s)	0.8 ± 0.1^1^	0.9 ± 0.1^1^	0.1 (−0.21, 0.13)^1^	0.9 ± 0.1^3^	1.0 ± 0.1^3^	0.1 (−0.02, 0.2)^3^	−0.02 (−0.1, 0.1)	0.705	0.1
SPPB (score 0-12)	10.4 ± 0.8^1^	11.3 ± 1.1^1^	1.0 (0.4, 1.6)^1^	10.9 ± 0.4^3^	11.6 ± 0.8^3^	0.7 (−0.2, 1.6)^3^	0.3 (−0.7, 1.2)	0.539	0.2
Average Hand Grip Strength (Kg)	29.5 ± 10.2^1^	32.0 ± 10.3^1^	2.6 (0.2, 4.9)^1^	28.2 ± 6.3^3^	29.3 ± 5.9^3^	1.1 (−0.3, 2.5)^3^	1.4 (−2.2, 5.1)	0.423	0.1
Average Stair Climb Time (sec)	5.4 ± 1.8^1^	5.0 ± 1.0^1^	−0.4 (−1.1, 0.4)^1^	4.4 ± 0.8^3^	4.3 ± 0.7^3^	−0.1 (−0.6, 0.5)^3^	−0.3 (−1.4, 0.9)	0.625	0.04

Baseline and 12-week values are mean ± SD

*SPPB *Short Physical Performance Battery, *Sec* seconds

Indicates significant difference in change between groups (*p*<0.05)

^1^n=16; ^2^n=8; ^3^n=7

## Discussion

This study systematically identified converging and diverging themes pertaining to participants’ experiences and perspectives towards supervised gym-based exercise and unsupervised home-based exercise. To our knowledge, this is the first mixed-methods study to explore the transition from a supervised gym-based HiRIT program, enforced by pandemic-related lockdowns, to an unsupervised home-based RT + AT program amongst older adults with obesity undergoing dietary weight loss. Participants’ experiences and perspectives on unsupervised home-based exercise were centralised around themes highlighting a lack of supervision, engagement, and equipment as well as a structured routine and no support from digital health modalities. Nevertheless, participants also emphasised support from healthcare professionals, accessibility, accountability, motivation and maintaining physical activity levels were critical to implementing unsupervised home-based exercise.

Some of the themes identified in the current study are similar to other qualitative studies exploring participant perspectives to gym- and/or home-based exercise interventions combined with dietary interventions [24, 39, 40]. A study in 10 older adults with chronic conditions (mean age: 65.8 years) investigated perspectives to gym- or home-based exercise interventions conducted over 12 months [24]. Participants in the home-based intervention reported enjoying the convenience of not having to travel to a centre to complete their intervention [24]. Likewise in our study, HOME participants identified accessibility as a key motivator for unsupervised home-based exercise and indicated that they were able to complete the program in their own time at their own convenience. This aligns with extensive research indicating that perceived convenience and autonomy are significant contributors to adherence in home-based exercise programs among older adults [41, 42]. A systematic review identified key factors associated with adherence to home-based exercise among older adults with chronic diseases [41]. They highlighted that accessibility to home-based exercise reduced logistic barriers such as transportation and scheduling conflicts, which often deterred participation in centre-based exercise programs [41]. Designing interventions that leverage convenience and personal autonomy are critical to promote sustained increases in physical activity among older adults with obesity.

Another study investigated the factors influencing adherence to home-based exercise in 10 stroke survivors in India (mean age: 61 years) [43]. They reported difficulties with motivation and commitment and a lack of access to professional supervision influenced adherence to home-based exercise [43]. In the current study some HOME participants identified they felt a lack of motivation to complete their prescribed exercises which stemmed from inadequate support and supervision leading to concerns of safety while completing home-based exercise unsupervised. Other HOME participants reported motivation as a dichotomous aspect of the program. Some participants expressed they were motivated to continue completing the exercise as they experienced positive changes from the program, while others indicated that the exercises tended to be monotonous, and they did not have the motivation to complete their prescribed program. These feelings of monotony towards the exercises may be attributed to the lack of in-person support and that the home-based RT + AT program was not as challenging, nor could it be progressed to the degree of HiRIT program. These findings are in line with previous research demonstrating that structured supervision and program progression are critical components of effective exercise interventions for older adults [44]. Ensuring a balance between autonomy and structured support may enhance long-term adherence and outcomes in this population.

A pertinent perspective highlighted in this study was the participants’ desire for the inclusion of digital health modalities for the delivery of unsupervised home-based exercise programs. Indeed, there is a growing recognition of technology as a modality in promoting engagement and compliance in home-based exercise interventions [45, 46]. In the current study implementation of digital modalities to support delivery of the unsupervised home-based RT + AT was prompted and discussed with study participants. Participants indicated digital health modalities may facilitate engagement, motivation and confidence and thereby improve adherence to the home-based intervention. Several studies have demonstrated that digital modalities may improve engagement and adherence to home-based exercise interventions [47, 48]. A follow-up qualitative study by Jansons et al. identified several themes including motivation, ease of use and enjoyability which supported the notion that home-based exercise programs delivered and monitored by digital modalities are feasible and pragmatic for older adults [47]. Despite their benefits, digital health solutions have potential limitations, such as challenges in usability and technological literacy among older adults, which must be considered when implementing such interventions [49, 50]. Additionally, completing unsupervised home-based exercise programs, even with the support of digital modalities may still be unsafe and unfeasible for some older adults. A systematic review and meta-analysis of 21 RCTs in community-dwelling older adults (aged ≥ 60 years) reported on the safety of and adherence to unsupervised home-based RT [51]. Of the 21 included studies, five studies reported intervention related adverse events and seven studies reported 38% of participants had 100% adherence to prescribed exercise programs (range 15–83%) [51]. These findings reinforce the importance of individualising home-based interventions to optimise safety and adherence for older adults. Tailored interventions that consider physical limitations and individual needs may enhance engagement and acceptability, while reducing safety concerns.

Although this study was not designed nor powered to detect between-group differences in functional outcomes, exploratory analysis revealed that changes in physical function did not differ between GYM and HOME in our study, but these outcomes did improve for GYM participants. A recent study examined the effects of home-based resistance training in 9 overweight (mean BMI: 26.0 ± 4.0 kg/m^2^) older adults (mean age: 68 ± 7 years) and also observed no changes in physical function outcomes [52]. Interestingly, participants in this previous study experienced a significant increase in total fat mass of around 5% [52]. Conversely, the only significant difference observed between groups in our study was a greater decline in fat mass for HOME compared with GYM. The reason for this difference is unclear, but a possible explanation is that HOME participants who were prescribed both RT and AT, may have adhered more closely to the hypocaloric diet than those required to attend the gym for the entire intervention. This could be due to the reduced time commitment associated with home-based exercise compared to attending structured gym sessions, potentially allowing for better meal planning, preparation, and overall dietary adherence. Further studies are warranted to investigate the effects of unsupervised home-based exercise on physical function and body composition in older adults with obesity transitioning from gym- to home-based exercise, particularly given participation in gym-based interventions decreases and/or ceases following the conclusion of a program supported and subsidised as part of a research intervention [53, 54]. Home-based exercise prescription may mitigate this decline in adherence by reducing costs and increasing accessibility to exercise [55–57]. A previous study in 105 older adults with chronic diseases randomised to home-based (*n* = 51; mean age: 66 ± 13 years) or gym-based exercise (*n* = 54; mean age: 68 ± 11 years) for 12 months [55] identified no differences in body mass, physical function or quality of life outcomes between gym-based and home-based exercise [55]. However, a recent meta-analysis investigating centre-based vs. home-based geriatric rehabilitation in older adults (aged ≥ 60 years) reported centre-based programs improved lower limb strength and timed-up and go scores to a greater degree than home-based programs [58]. While structured and supervised programs may yield superior functional benefits, particularly for strength-based outcomes, unsupervised home-based programs may be a suitable alternative to maintain physical activity levels in older adults with obesity.

### Limitations

A limitation of this study was a lack of objective measurement of adherence to unsupervised home-based exercise in the 13 participants that were forced to transition to home-based exercise due to COVID-19 lockdowns. Furthermore, the GYM and HOME programs were not comparable in terms of exercise intensity as a lack of equipment and supervision limited the ability for participants to safely complete HiRIT. Finally, the analysis of quantitative outcomes was exploratory and thus not powered to detect significant between-group differences for changes in body composition and physical function measures, as such, effect sizes were used as a more appropriate measure of outcomes.

## Conclusions

This study identified pertinent experiences and perspectives associated with transitioning from supervised gym-based to unsupervised home-based exercise interventions in older adults with obesity undergoing weight loss. Participants’ experiences and perspectives on the home-based program included its accessibility and the sense of accountability in completing the exercise. However, some participants experienced challenges such as limited support and supervision while completing the exercises at home, as well as insufficient access to the necessary equipment to complement the intervention. Additionally, digital health interventions may support remote delivery of, and participants’ confidence in completing, unsupervised home-based exercise interventions. Unsupervised home-based exercise alongside caloric restriction was associated with significantly greater decreases in fat mass and similar changes in physical function compared with gym-only exercise. However, ensuring adherence and long-term engagement in home-based exercise programs remains a critical challenge that requires personalised interventions and structured support for older adults with obesity. Further research is needed to better determine methods to support transitioning from supervised gym-based exercise to unsupervised home-based exercise for older adults with obesity and its effectiveness for improving body composition and physical function.

## Supplementary Information

Supplementary Material 1

Supplementary Material 2

Supplementary Material 3

## Data Availability

The datasets used and/or analysed during the current study are available from the corresponding author on reasonable request.

## Abbreviations

APD: Accredited Practising Dietitian
AT: Aerobic Training
COREQ: Consolidated Criteria for Reporting Qualitative Research
DXA: Dual Energy X-ray Absorptiometry
HiRIT: High Intensity Resistance and Impact Training
SPPB: Short Physical Performance Battery
RCT: Randomised Controlled Trial
RM: One Repetition Maximum
RT: Resistance Training
