# Supplementary material for: Experiences and outcomes of older adults with obesity transitioning from gym- to home-based resistance training due to COVID-19 lockdowns: a mixed-methods analysis of a RCT
Source: BMC Geriatr. 2025 Jul 29;25:556. doi: 10.1186/s12877-025-06247-3 (PMC12309126; doi:10.1186/s12877-025-06247-3)
Supplement: Supplementary file 1 — Supplementary Material 1 [file 12877_2025_6247_MOESM1_ESM.docx]

**Supplementary File 2.** Qualitative Interview Questions

1. Can you tell me why you decided to take part in this study?
2. What, if any, have you found the benefits of the gym-based exercise program to be?
3. What, if any, have you found the negatives of the gym-based exercise program to be?
4. What, if any, have you found the benefits of the home-based exercise program to be?
5. What, if any, have you found the negatives of the home-based exercise program to be?
6. How easy or difficult was it for you to regularly participate in your prescribed home-based exercise program during a Covid-19 lockdown/s?
7. Do you believe that the prescribed home-based exercise program was a good substitute in place of supervised gym-based exercise? Why/why not?
8. How could we best support you to participate in your osmosis program during Covid? For example, would you find it helpful to receive support using ‘telehealth’ options such as phone calls, text messages, video conferencing, videos of the exercises.
9. Do you see any advantages or disadvantages of home-based exercise using telehealth methods like this compared with supervised gym-based exercise programs?
10. Have you continued exercising since the study finished, or do you intend to do so? If so, have/will you be doing gym-based exercise, home-based exercise, or a mixture of the two and why? If not, why have you stopped?
11. Do you have any further comments regarding this study?
