# Supplementary material for: Experiences and outcomes of older adults with obesity transitioning from gym- to home-based resistance training due to COVID-19 lockdowns: a mixed-methods analysis of a RCT
Source: BMC Geriatr. 2025 Jul 29;25:556. doi: 10.1186/s12877-025-06247-3 (PMC12309126; doi:10.1186/s12877-025-06247-3)
Supplement: Supplementary file 2 — Supplementary Material 2 [file 12877_2025_6247_MOESM2_ESM.docx]

# **SUPPLEMENTARY MATERIAL**

**S1.** Qualitative themes and subthemes with illustrative quotes.

| **Theme** | **Illustrative Quotes** |
| --- | --- |
| Accessibility | “But I did that religiously because we were in lockdown.” (Participant 30, female, age 70).  “Because we were locked down, it was, basically, one of the things that you could do” (Participant 30, female, age 70).  “Yeah. It's just that it-- that it's just too hard to get to a gym here 'cause they're-- none of them are really local. It would be like 45 minutes to any one of them.” (Participant 48, female, age 64)  “You know. But what worked better was if I had a-- if I was outside and I had a minute spare, I'd do a push-up against the fence, or I'd-- or I'd, um, do my toe raises, you know, or my sitting up and down.” (Participant 49, female, age 63). |
| Accountability | “I'd have to report in every week and take a photo of my, um, exercise diary.” (Participant 30, female, age 70).  “So I had to fill in an exercise diary and then, you know, photograph it and send it in, email it off to him. And then he'd check it, and then he'd, he'd, uh, increase it. Like he'd either increase the reps or increase the weights” (Participant 30, female, age 70).  “So that, you know, you're sort of-- you, you feel like, ‘Okay. You know, I'm still-- I'm still accountable, to some degree.’ And as well, you know, you still have the interaction and the, um, you know-- and, and the option to sort of-- for someone to say, you know, ‘So how's it going? Are you finding it difficult? You know, any way we-- you know, anything you think we can do to help more?’” (Participant 48, female, age 64).  “Just keeping you accountable I suppose-- just-- really. 'Cause you make this commitment, um, and, you know, we should be-- you know, the participants should be accountable for it, otherwise they don't want to do it.” (Participant 54, male, age 68)  “Yeah. I think so. I think, you know, if we-- if we made a commitment to the, um-- for the gym guy-- the actual gym attendant, then keep that commitment to do the exercise at home. Because I think, you know, most people would've allocated that time.” (Participant 54, male, age 68). |
| Support from healthcare professionals | “And I had the backup and support of, of the people there at Monash” (Participant 30, female, age 70).  “I was also supported because I could contact Jake any time to ask any questions” (Participant 30, female, age 70).  “Um, because then, at least I felt like, "Okay. Well, I can't cheat on this, you know. I have to do it, and I have to do it-- I have to do the exercises twice a week, and I have to do the walking three times a week-- because I have to fill in the form and give it to you.” (Participant 48, female, age 64)  “Um, yeah. Well, you sent me notes and asked me how I was going, so I did, uh, I stayed on it. But, um, uh, that was, that was good. But, um, yeah. So I'm fairly self-motivated. I guess I was, uh-- if I was less motivated, I might have needed, um, you know, somebody to call in on the morning to see if I was going to do it. But, um” (Participant 52, female, age 63). |
| Maintaining Physical Activity Levels | “Gave me the opportunity to maintain my strength and ability to, you know, go back to the weights without, you know, without having to start over.” (Participant 48, female, age 64).  "I think the things that you gave me were, you know, were pretty good. And I think every time I went back to the gym, I hadn't-- you know, I hadn't regressed, which was good.” (Participant 48, female, age 64).  “And they make a difference. I mean, it's interesting that they, you know, in whatever it takes, I mean, 20 minutes or so, you, you really do build up a sweat. Um, yeah. It's quite interesting.” (Participant 52, female, age 63).  “I certainly do. I feel much more stable, uh, you know, and I don't feel like I'm waddling and like I-- you know, my, sort of my stomach has, has shrunk, so I feel, um, like with my squats I'm much more able, um, to do them. Ah, yeah. The only thing I would say is that it's, it is-- I still do find my, uh, knees for whatever reason still bother me a little when I do the squats. So, um-- but overall, great. Great positives, yeah.” (Participant 52, female, age 63) |
| Motivation | “Well, absolutely. As I just said, it was-- it really motivated me.” (Participant 30, female, age 70).  “And also, because I felt better, uh, it-- I could feel myself getting better each week, it motivated me more.” (Participant 30, female, age 70).  “So, um, yeah. So it was, it was good to do the home-based ones because you get into a routine and so you can, you know, you'll continue that, potentially, after the study.” (Participant 52, female, age 63). |
| Openness to telehealth videoconferencing for support | “I, I think that was good to have that there on hand all the time for me to use, but I, I think, um, yeah, telehealth or video conferencing would be even more beneficial.” (Participant 30, female, age 70)  "Zoom conference would have been really good actually, especially if there was, um, you know, more than one.” (Participant 44, female, age 76).  “I think it's more, you know, like just either giving some direction and then letting the person go off and do it or letting the person do their exercises and then checking in and having a, a, you know, a two-way conversation on a Zoom call or whatever just to have a chat and see how it's going and report back. I think, to me-- for me personally, that-that's probably the better option.” (Participant 48, female, age 64).  “I think it depends on your ability to, um, organise your life. I think some people would've benefitted a lot by a Zoom contact once a fortnight” (Participant 49, female, age 63).  “Keep that commitment, but then have a, you know, a Zoom or a teleconference or something to say, ‘Okay. Let's, let's run through it and let's see how you're going.’"(Participant 54, male, age 68). |
| Lack of equipment | "So that, that, that would be a disadvantage, I suppose, not having the equipment.” (Participant 26, female, age 67).  “I mean, obviously, it was difficult to replicate the weights” (Participant 48, female, age 64).  “Um, well, only that I just-- because I didn't have, um, the equipment, I probably didn't full-- do the weights to the, you know, the weight as heavy as I was doing, and so I wasn't able to progress.” (Participant 52, female, age 63)  “And if I was another participant and I didn't have the weights, I think that it would not have been a good, good substitute.” (Participant 52, female, age 63).  “Um, well, have-- as I say, having weights makes it-- makes a difference because it, it just really does build the strength.” (Participant 52, female, age 63).  “Yeah. The home-based exercise, it's pretty hard when you don't have access to, to those weights” (Participant 53, female, age 70). |
| Lack of supervision | " You're there, and they're watching you directly and supervising you, so I think obviously, that's going to be better.” (Participant 30, female, age 70).  “Like in the beginning, I, you know, was emailing and sending off my weight, and got no responses. And so one week, I knew I did the wrong thing and I thought, "Well, I won't send my weight in," and I didn't get a reaction. So the following week, I didn't do it again and didn't get a reaction and in the end I thought, "Well, there's a huge lack of interest there." You know, and so if someone's not motivating me-- that's why I go to the gym. And I do prefer to take part in organised classes” (Participant 53, female, age 70).  “Well I didn't-- I, uh, didn't think-- there was no, um, uh, oversight, because, obviously, it was left up to me to do. And sometimes it was left up to me to do, I'd take a very laissez-faire attitude towards it.” (Participant 54, male, age 68).  “Um, A, because I didn't feel that I was getting enough value from it-- [you know?] physically getting enough value or improving sufficiently from it. Um, and B, I needed that sort of-- I needed you to, to push me and to-- and the commitment of you've got to be there at this time on this day, so.” (Participant 54, male, age 68). |
| Lack of engagement | A couple of participants identified they could not engage with the home-based program, stating they found it boring and monotonous.  “Yeah. But the repetitions, you know, doing 30-odd, what was it, calf raises or something, and-- You know, it, it is monotonous doing it. Yeah.” (Participant 53, female, age 70).  “Oh, boring as [laughter]. Pretty boring.” (Participant 53, female, age 70).  "Well [inaudible] I didn't, because I, I didn't really feel that they were-- the exercises were strong enough. Does that make sense?” (Participant 54, male, age 68).  “Uh, one, one of the exercises was just standing up-- standing on your toes and lifting up and down a bit. And I found it quite easy to do. And I thought, ‘Um, I don't know what benefit I'm getting.’ I couldn't feel I was getting any benefit out of it.” (Participant 54, male, age 68). |
| Lack of structured routine | "Without having the commitment of, you know, of having to meet-- you know, having an appointment to meet someone else, I found it harder to make the time for it.” (Participant 48, female, age 64).  “I work strange hours. You know, um, running a house and stuff, it-it's sort of hard to find time for yourself” (Participant 48, female, age 64).  “I hadn't made that a priority. So having the appointments where, you know, obviously, I had an appointment, I had to be there at that time was a little more, um-- a little more controlled than me, ‘Okay. You know, w-- uh, w-what time, you know, will I do the exercises today or tomorrow?’ and, you know, ‘What time will I do it?’ Cause it was easy for that-- you know, for the commitment that I'd made to myself to slip, and then I'd have to, you know, catch up and say, Okay. Gee, if I don't do it today, then I'm gonna-- you know, I'm gonna miss out. Um, I won-- I wo-- I won't be able to meet the requirements for this week.’” (Participant 48, female, age 64).  “Yeah. Yeah. Like every day I'd think, "Oh, I've got to do those. I'll do them in an hour. No, I'll do them in half an hour." Sometimes it was like 3:00, 4:00 before I got around to it.” (Participant 53, female, age 70). |
